# Hepatitis E Virus and Renal Transplantation

**DOI:** 10.5812/kowsar.1735143X.770

**Published:** 2011-08-01

**Authors:** Seyed Mohammadmehdi Hosseini Moghaddam

**Affiliations:** 1 Transplant Infectious Diseases, University of Toronto, Toronto General Hospital, Toronto, Canada

**Keywords:** Hepatitis E virus, Renal transplantation

Sepehrvand et al. demonstrated a considerable seroprevalence rate of anti-HEV in Iranian kidney transplant recipients [1]. The impact of HEV infection on renal transplantation and the risk of chronic HEV infection in this group are debated-issues that I would like to discuss. Hepatitis E virus (HEV) was first discovered in New Delhi, India, in 1955 [2]. The virus is transmitted via the oral-fecal route [3]. Other possible routes of transmission include blood transfusions, drug vertical transmission, person-to-person contact, and zoonotic transmission [4][5]. The frequency of HEV transmission by non-fecal-oral routes remains unknown [2][6]. In endemic areas, exposure occurs in childhood [7][8]. In high-income countries, most cases of hepatitis E appear to be acquired locally and are not imported from endemic regions. In these areas, it likely has a zoonotic origin [9]. In immunocompetent individuals, hepatitis E is a self-limited disease. However, HEV can cause chronic infection in solid organ transplants [10][11], patients who receive chemotherapy [12], and HIV-infected persons [13]. HEV infection causes chronic hepatitis in more than 60% of recipients of solid organ transplants. Factors that increase the risk of chronic hepatitis in solid organ transplant recipients are shorter interval since the transplant, lower levels of liver enzymes and serum creatinine, lower platelet counts, and tacrolimus-based immunosuppression (compared with cyclosporin A), the most significant of which are tacrolimus use and low platelet count [11][14][15].

In otherwise healthy kidney transplant recipients, HEV might be considered the etiological agent for the development of hepatitis in those who live in endemic regions [16]. Viral hepatitis E may progress rapidly to cirrhosis in renal transplant recipients [17]. Although occult infection of HEV may be transferred to the recipient via liver graft [18], other allograft organs appear to be clear for transmission of the virus. As a result, screening for HEV at the time of transplantation is only recommended in liver transplant donors and recipients in endemic areas [19]. Such a screen is not recommended for patients with renal failure who are waiting for renal transplantation. Rising liver enzyme levels is a nonspecific finding following solid organ transplantation. Renal transplant patients may experience such increases, due primarily to drug reactions, sepsis, and hepatotropic virus-related infectious diseases. The diagnosis of viral hepatitis E in renal transplant recipients is usually made by ELISA. In renal transplant recipients, the seroprevalence of anti-HEV IgG is 6% to 16% [10]. Other immunocompromised hosts, such as patients with hematological malignancies, might avoid forming HEV IgG following an infection. Moreover, viremia may exist for more than 6 months after an acute infection [20]. In addition, the development of HEV IgG in renal transplant patients does not appear to be universal. The presentation of chronic hepatitis in renal transplant patients may be associated with normal liver enzymes and a negative serological assay [21]. This phenomenon underscores the need for molecular studies in suspected subjects.

Decreasing the numbers and doses of immunosuppressive drugs remains the first approach toward controlling viral hepatitis E in renal transplant recipients. A prolonged follow-up period might be required to assess the eventual outcome [14]. In addition, pegylated interferon alpha-2b may be useful in the management of chronic HEV infections in solid organ recipients in whom a reduction of the immunosuppressive regimen is insufficient [22]. Interestingly, 3-month Peg- IFN-α-2a therapy was shown to be efficacious in a hemodialysis patient with chronic HEV infection following renal transplantation [23]. In 2010, the efficacy of ribavirin 12 mg/kg of body weight daily for 12 weeks was reported in kidney transplant patients with chronic HEV infection. However, due to the short term follow-up (3 months), eradication of the virus could not be claimed [24]. In 2011, another report demonstrated that a 3-month course of oral ribavirin (17 mg/kg/day) in solid organ transplant patients with chronic HEV infection induced a sustained virological response for more than 4 months after cessation of ribavirin [25]. A long follow-up is always required to evaluate the outcome of HEV infection in solid organ transplant patients. HEV infection may cause cirrhosis in renal transplant individuals. As a result, close follow-up is required after the diagnosis. In addition, HEV infection may result in nonhepatic complications in kidney transplant recipients. Neurological diseases that affect the peripheral or central nervous system have been demonstrated in renal transplants with chronic HEV infection. Surprisingly, HEV was isolated from the cerebrospinal fluid in such patients [26].
